# Public–private partnerships for universal health coverage? The future of “free health” in Sri Lanka

**DOI:** 10.1186/s12992-019-0522-6

**Published:** 2019-11-28

**Authors:** Ramya Kumar

**Affiliations:** Department of Community and Family Medicine, Faculty of Medicine, University of Jaffna, Jaffna, Sri Lanka

**Keywords:** Sri Lanka, Healthcare access, Universal health coverage, Health systems, Health reform, Healthcare privatization, Public–private partnerships

## Abstract

Sri Lanka reports impressive health indicators compared to its peers in the South Asian region. Maternal and infant mortality are relatively low, and several intractable communicable diseases have been eliminated. The publicly financed and delivered “free” healthcare system has been critical to these health achievements. Placing the country’s healthcare system in historical context, this commentary analyses the contradictions and political tensions surrounding Sri Lanka’s 2018 Universal Health Coverage (UHC) policy, with attention to the Ministry of Health’s plans for public–private partnerships (PPP). As economic exigencies and private interests increasingly erode the 1951 “Free Health” policy, this commentary calls for a re-envisioning of UHC that can meet people’s aspirations for health and social justice.

## Background

More than three decades after the release of the Rockefeller Foundation’s Bellagio Conference report, *Good Health at Low Cost* [1], Sri Lanka continues to report impressive health indicators. It has the lowest maternal mortality ratio in the South Asian region: 30 maternal deaths per 100,000 live births in 2015, as compared with Bangladesh (176), India (174), Maldives (68), Nepal (258) and Pakistan (178) [2]. In addition, Sri Lanka has achieved elimination status in the control of several intractable communicable diseases, including poliomyelitis, malaria, and, most recently, measles [3–5]. In 2018, WHO celebrated World Health Day in Colombo, marking the country’s accomplishments in healthcare coverage [4].

Sri Lanka’s acclaimed publicly financed and delivered “free” healthcare system is widely acknowledged as a critical factor underlying its health achievements [6–8]. Guided by a “Free Health” policy (1951) adopted following independence, the public healthcare system comprises state-financed and administered healthcare facilities that remain free of charge at the point of use, covering about 50% of outpatient services, 90% of inpatient admissions, and nearly all preventive services. Financing has remained tax-based within the public system, with no separation of purchasing and provision [6, 8]. In fact, a 2018 World Bank-commissioned study highlighted the country’s rejection of orthodox health-financing reforms [6].

On the other hand, a fast-growing private health sector flourishes in parallel to the public system, accounting for over half of national health expenditures, much higher than its contribution to actual healthcare delivery. In 2015, 54% of health spending came from private sources: 85% of this paid out-of-pocket, 5–8% comprised employer benefits, 5% health insurance, and 2–3% from the non-profit sector [9]. Despite 40 to 45% of total health spending being financed out-of-pocket, catastrophic and impoverishing health expenditures have remained comparatively low because the public system still covers the bulk of (more expensive) inpatient care [6, 10].

The ever-expanding for-profit private sector fills a critical gap in public-sector ambulatory services. As state investment in the public system is grossly inadequate to address the demand for health services, users are compelled to pay out-of-pocket for services from the private sector. With an escalating burden of non-communicable diseases (NCD), and a rapidly aging population, the burden of out-of-pocket expenses is expected to rise further [11, 12]. In October 2018, the Ministry of Health adopted a new policy, “Healthcare Delivery for Universal Health Coverage” [13], setting out expansive healthcare reforms to improve population coverage, financial risk protection, and service comprehensiveness. Reforms aimed at streamlining health-service delivery in the public sector include plans for contracting-in private providers to improve healthcare coverage. As Sri Lanka now embarks on reforming its long-acclaimed “free” healthcare system, this article questions public financing of private delivery as a path to achieving UHC.^1^

## Beginnings of “free health”

The foundations of the Western medical system in Ceylon (now Sri Lanka) were laid in the nineteenth century under British colonial rule. The hospital system, set up through missionary and philanthropic efforts, expanded when missionary hospitals were brought under the Civil Medical Department (1858) [14, 15]. The health units system—introduced by the Rockefeller Foundation in the 1920s—laid the foundations for a hugely successful preventive health sector [16]. Rural expansion of healthcare accelerated after the 1934–1935 malaria epidemic when the anti-colonial Suriya Mal movement^2^ became active in malaria relief efforts [17]. By the time of independence (1948), the public system comprised about 250 institutions spread across the country [18].

The “Free Health” policy was adopted in 1951. Prompted by the 1946 legislation of Britain’s National Health Service, the government commissioned the Cumpston Report (1950), which set the direction for health reform in post-independent Ceylon [15]. User-fees were abolished in the public system, and state services were brought under a centralized health department [15, 19]. Arguably more important for the country’s health achievements were a series of social policies adopted in the wake of universal franchise (1931) in the late colonial period [1, 20]. Food subsidies came into effect after a food-rationing scheme was introduced during World War II. “Free Education” was adopted in 1945, granting universal primary and secondary education [20]. Although these policies were gradually chipped away over subsequent decades, their legacy endured, shaping demands on the state, as demonstrated in the 1953 trade-union-led countrywide protest against axing the rice subsidy [20, 21].

As terms of trade became unfavorable to the country’s export-dependent economy, the government—under International Monetary Fund (IMF) and World Bank pressures—began to cut back on welfare [21, 22]. However, state investment in the health sector remained high through the 1960s. There were no major reforms to the healthcare financing system, apart from the introduction of a stamp duty for outpatient services (1971), subsequently removed by the incoming 1977 government [19, 23]. Having signed on to an IMF agreement supporting trade liberalization, currency devaluation, removal of price controls, welfare cuts, and privatization [21], the government began a long-term, incremental project of dismantling the public healthcare system through sustained underinvestment and incentivized private expansion [7, 23].

## Private-sector incursion

Private healthcare services have operated in parallel to the public system since its early days, when the British colonial government encouraged private practice among public-sector specialists to maintain low wages for physicians in the public system. As dual practice created a channel through which private patients gained entry to government hospitals [15], it paved way for a private sector that became reliant on the public system for human resources. Even today, most private-sector doctors, whether specialists or general practitioners (GPs), hold full-time positions with the Ministry of Health [24].

Successive governments in the post-independence period attempted to restrict private practice. A ban was imposed in the 1950s on newly qualifying public-sector specialists and medical officers engaging in private practice. The government implemented the ban quite loosely, and the 1960s saw public-sector specialists regaining some private-practice privileges as a result of strike action by the public-sector physicians’ union. Specialist private practice was subsequently permitted at designated state-administered centers which generated revenue for the government. In the 1970s, the leftist alliance in government attempted to phase out dual practice by prohibiting such practice at stations where specialists were available full-time in the private sector. Implementation of this policy resulted in specialists leaving state service en masse, to work full-time in the private sector. In 1977, with economic liberalization, these restrictions were removed; dual-practice privileges were extended to general doctors and, later, other categories of health professionals [25].

The private health sector grew steadily through the 1980s and 1990s, although the availability of resource-intensive tertiary care was limited until the Board of Investment extended its privileges to the healthcare industry by offering tax holidays, concessionary rates on corporate income tax, import-duty exemptions, and concessionary lease terms on state lands [26]. Several large-scale private hospital projects were approved by the Board of Investment, changing the landscape of private healthcare, particularly in the commercial capital, Colombo [27]. The small private-health insurance market has expanded steadily, its contribution to private health spending rising from 1 to 5% between 1990 and 2009 [9]. The end of the civil war in 2009 meant a major boost for the private health sector, facilitating expansion in the ensuing decade.

## The current status of UHC

As defined by the United Nations, UHC encompasses provision of essential healthcare services of high quality to all without financial hardship [28]. This section maps the structure and organization of healthcare in Sri Lanka, highlighting the relative contributions of public and private healthcare to the components of UHC: financial risk protection, population coverage, and comprehensiveness of service coverage.

Sri Lanka’s healthcare system consists of a dominant tax-funded public system supplemented by a fee-for-service private sector. In 2016, total health spending accounted for just 3.9% of the Gross Domestic Product (GDP), translating to a per capita expenditure of USD 153 [2]. According to 2015 estimates, 54% of total expenditure on health is financed by private sources—some 85% of this out-of-pocket [9].

### The public healthcare system

The most remarkable feature of Sri Lanka’s public healthcare system is that it offers services on a walk-in basis with no charges at points of use.^3^ Financed chiefly through (regressive) indirect taxation, the system has achieved impressive geographic spread at relatively low levels of spending (< 2% of GDP) [6, 8, 9]. However, underinvestment in the system has resulted in widespread shortages in human resources, medical supplies and services, as well as inequitable service distribution [6, 7, 24, 29].

The central Ministry of Health and nine provincial health departments administer public healthcare services. The central Ministry oversees apex referral centers, runs disease control programs (Anti-Malaria Campaign, National STD/AIDS Control Program, etc.), provides technical guidance, and undertakes policymaking, human resources training and recruitment, purchasing and distribution of drugs and medical supplies, and research and development. Apart from a small share of primary care services administered by municipal authorities, the provincial departments of health administer all other healthcare facilities, including the bulk of preventive care [6, 7, 24].

Preventive and curative sectors operate separately in the public system. Preventive service delivery is decentralized to the provincial departments of health through 26 health regions, made up of ~ 350 Medical Officer of Health Areas covering the entire country. Each Medical Officer of Health —a general practitioner with public health training—is supported by a public health team comprising assistant medical officers of health, public health nurses, public health midwives and public health inspectors. The Medical Officer of Health Areas (1:∼50,000 population) are further divided into Public Health Inspector Areas (~ 1:10,000 population), which, in turn, are divided into Public Health Midwife Areas (1:~ 3000–5000 population) [30].

Maternal and child health (MCH) is a key focus of the Medical Officer of Health system. Clinic-based and domiciliary care is delivered by public health teams, while an effective referral system links field services with hospital-based specialist care [30]. MCH services target married women of reproductive age and children < 5 years [30], raising accessibility concerns for persons outside traditional family structures. The Medical Officer of Health also supervises delivery of school health services, screening for NCDs, food and environmental sanitation, and occupational health services [30, 31].

Communicable disease control activities are coordinated by the preventive health sector. A comprehensive immunization program protects children against vaccine-preventable diseases [32]. A surveillance system links Medical Officer of Health Areas with district-level and central administrations. The control of several infectious diseases, and the elimination of polio (2014), malaria (2016) and measles (2019), are credited to this system [3–5].

Access achievements are impressive in the preventive health sector. Antenatal coverage and skilled attendance at birth are an impressive 98% with 94% of deliveries taking place at public facilities [33]. Over 90% of those under three years have been immunized against twelve infectious diseases [33]. Although MCH services and communicable disease control activities are well established in the preventive system, services for NCDs are still in nascent stages of development [34].

In contrast to the preventive health sector, which systematically covers the entire country, the curative sector has developed in an ad hoc manner [35]. In 2018, the latter comprised 1105 facilities, spanning primary, secondary, and tertiary levels [36]. Notably, the distribution of tertiary care is inequitable, with rural, plantation, and war-affected areas faring worse. For instance, in 2017, 193 of 514 (37%) cardiology beds in the public sector were in the Colombo District, with the remainder spread across 11 health districts; 14 districts—seven of them in war-torn regions—reported zero cardiology beds that year [29].

The primary curative care system is under-resourced and weak. Most primary care centers, especially those in rural and remote areas, are understaffed and experience recurrent shortages in drugs and medical supplies. Without empanelment, primary-care centers in the curative sector provide little continuity of care. These service deficits result in users bypassing primary-care facilities for overcrowded secondary or tertiary centers [12]. These gaps in curative care are targeted by the 2018 UHC policy [13, 37].

The public health sector is staffed by healthcare workers trained on a non-fee levying basis under the “Free Education” policy. The Ministry of Health trains nurses, midwives, and other ancillary health professionals, with many categories receiving stipends during the training period. Physician training takes place at non-fee levying state universities,^4^ and the Ministry of Health subsidizes postgraduate training for its specialist cadre. Most categories of healthcare professionals are guaranteed full-time salaried employment in the public sector. They are required to serve in remote areas to gain promotion, ensuring a widely dispersed healthcare workforce, albeit with considerable regional disparities. In 2017, 620 specialists, 3093 hospital medical officers and 8562 nurses were serving in the Colombo District, compared with only 147 specialists, 782 hospital medical officers, and 1234 nurses in all five districts of the war-torn Northern Province [29]. Admittedly, the post-war Northern Province is sparsely populated, accounting for just 5.2% of the country’s population compared with 11.3% in Colombo District. Even so, human resource disparities are significant. Service requirements in remote areas, inflexible transfer procedures, and the availability of employment options in the ever-expanding private sector and abroad, all promote brain drain from the public sector [27].

### The private health sector

The private sector comprises an ad hoc range of healthcare facilities, from GP clinics and specialist consultation centers to smaller-scale in-patient facilities and large commercial hospitals [7, 38]. These facilities are supported by numerous private pharmacies and diagnostic centers of varying standard and quality [39]. The non-profit sector plays a very small role in healthcare delivery, contributing mostly to contraceptive service provision, and addressing other service gaps, including for people with disabilities. A few cooperative hospitals and faith-based organizations offer a limited set of healthcare services [7].

Out-of-pocket payments are the primary barrier to accessing private healthcare. A greater share of out-of-pocket spending goes toward outpatient consultations, except in the highest wealth quintile, where spending on inpatient care is substantial [40]. The President’s Fund, a humanitarian initiative under the auspices of the President, offers (limited) financial assistance for a predefined set of resource-intensive procedures in the private sector, including cardiac, renal, and orthopedic surgery and cancer therapy [41]. As the Fund does not cover outpatient care and provides capped disbursements for inpatient care, economically disadvantaged healthcare users often cannot access these benefits.

A national health insurance scheme has not been introduced to date in Sri Lanka. A contributory health insurance scheme covers a specified set of health benefits for some public-sector employees [42]. Since 2017, a publicly-financed school health insurance scheme covers all students, with caps on claims [43]. A small but growing private health insurance industry covers mostly private sector employees with pre-paid insurance plans accounting for about 5% of private expenditure. Certain companies offer reimbursement schemes, but, taken together, employers contribute less than 10% to private spending [9].

Private healthcare services are available on a walk-in basis. GPs—many of them public-sector physicians working after hours—operate from independent clinics, spread across urban and rural settings. A specialist opinion may be obtained fairly easily at a specialist consultation center, with no requirements for referral. However, private specialist services tend to be concentrated in urban areas. As most private practitioners are employees of the Ministry of Health, users move easily between sectors, opting for private out-patient care, and turning to public hospitals for (more expensive) in-patient treatment [7].

The commercial hospital industry is mostly confined to Colombo and larger cities where wealth is concentrated. A major proportion of the private healthcare market, as much as 75% according to some estimates [27], is concentrated among four or five healthcare firms operating out of Colombo. This distribution of private healthcare has led to markedly different utilization patterns. For instance, in 2016, 21% of deliveries in Colombo District took place in the private sector, compared with a national average of < 6% [33]. This form of urban-centric private health sector development has implications for UHC as healthcare professionals increasingly opt for full-time private practice, causing a dearth of human resources for healthcare in rural areas [27].

### Quality concerns

Standard setting and regulation are critical aspects of quality assurance in healthcare. In the public sector, standard setting is under the purview of the central Ministry of Health with contributions from independent professional medical bodies. Monitoring and evaluation of public (and private) healthcare services are restricted by poorly developed health information systems [6].

Accreditation and licensing requirements are lax in the public and private health sectors. There are no Continuing Medical Education requirements for renewal of medical and other health professional licenses. While the maintenance of professionalism, discipline, and ethical medical practice is under the purview of the Sri Lanka Medical Council, mechanisms in place to redress medical malpractice are time-consuming, expensive, and lack transparency [44].

The private health sector is weakly regulated. In 2006, the Private Medical Institutions (Registration) Act transferred regulatory authority from the Ministry of Health to the Private Health Services Regulatory Council. With wide representation from the healthcare industry, the Council has been unable to implement the most basic requirements for registration [38]. Weak regulation manifests in dangerous forms of private practice and escalating out-of-pocket spending [45, 46]. The proposed UHC reforms aim to improve health sector accountability, through citizen engagement, and strengthen regulation of the private sector [37].

In sum, the public sector delivers a greater share of services within Sri Lanka’s mixed healthcare system. Accounting for less than 2% of GDP, it offers remarkable financial risk protection and population coverage at relatively low cost, albeit with accompanying deficits in human resources, medical supplies, and services, and inequities in access to healthcare. The private health sector, which contributes far less to service delivery, accounts for over half of total health expenditure. Although out-of-pocket payments restrict access to resource-intensive inpatient services, the private sector fills a critical gap in ambulatory care. Weak mechanisms of accountability undermine both systems, with private-sector regulation a major concern.

## Sri Lanka’s 2018 UHC policy

In October 2018, the Ministry of Health adopted a new policy, “Healthcare Delivery for Universal Health Coverage,” aiming to “ensure universal health coverage to all citizens, relevant to the disease burden experienced in the country through a well-integrated, comprehensive and efficient health service” (p.2) [13]. Conceived through a top–down stakeholder consultation process, and led by the Ministry of Health with support from the World Bank (and other “development partners”), the new policy seeks to reorganize primary healthcare and offer an essential services package through a “shared care cluster” model [37].

“Shared care clusters” refer to units made up of an apex public-sector specialist care center and its surrounding primary-care facilities. Unlike the current system, where users access their preferred public facility irrespective of residence, referral pathways will direct empaneled users in demarcated catchment areas to enter the system through their designated primary-care centers [37]. An essential services package spanning primary through tertiary care will be introduced to improve comprehensiveness of service coverage within clusters [36]. Key aspects addressed by the UHC policy are human resource strengthening, improving health information systems, community empowerment, and, notably, private sector engagement [13, 37].

This reform will require massive state investment in the health sector. At the primary-care level, existing health-worker cadres will be expanded (including a family doctor per 5000 population), several new categories of healthcare workers are to be recruited to multi-professional primary-care teams, and private GPs are to be contracted-in to improve geographical coverage and extend service hours. In terms of infrastructure, primary-care centers are to be equipped with emergency rooms, ambulances, and on-site laboratory and pharmacy services. In addition, private diagnostic centers and pharmacies will be contracted-in to address service deficits. A revamped health information system will enable tracking individual health records to improve continuity of care and minimize duplication of services. These plans will be implemented alongside measures to better integrate preventive with curative care and strengthen specialist services at secondary and tertiary care centers. In parallel, monitoring and evaluation, regulation, improved mechanisms of accountability, and citizen engagement are expected to improve the quality of care [13, 37].

Many of these policy directives, if implemented, will address critical gaps in Sri Lanka’s healthcare system. Streamlining referral pathways will increase utilization of primary care facilities and reduce congestion at secondary and tertiary care centers. Implementing an essential services package will improve service comprehensiveness, population coverage, and financial risk protection, particularly in underserved areas, and as regards deficit services for NCDs, mental illness, disability, and seniors. Apart from the knotty question of financing these reforms, which remains unaddressed in publicly accessible policy documents, the Ministry of Health’s plans for “effective engagement of the private sector” (p. 4) [13] lack clarity and direction.

## Public financing of private healthcare: contradictions and political tensions

The “Free Health” policy still enjoys wide public appeal in Sri Lanka [7]. As health economist William Hsiao highlighted nearly two decades ago, healthcare is a politically contentious issue in Sri Lanka “so much so that [user-fees] will not be officially debated in public” (p. 57) [35]. “Free Health” is frequently endorsed by politicians at all levels. For instance, the incumbent Minister of Health spoke of “Free Health” at the 2018 World Health Day celebrations in Colombo in this way:

Sri Lanka has a state funded and run health care system providing health care, free of charge at the point of delivery, to each and every citizen. The health budget is funded by the public sector, has a pro-poor health service that facilitates access to lower socio-economic strata of society. With a wide network of facilities … the Government provides a comprehensive package of services to the population. In spite of this situation, patients, especially those in the private sector, have to bear significant out of pocket expenditures …. To address this situation we have taken action to reduce the price of essential, mostly used drugs.... We have also lifted the price ceiling on cancer drugs to be provided free at the government facilities. … We have also extended similar benefits towards eye care, providing quality eye lenses to patients free of charge. Similar packages have been introduced for expensive, urgently needed cardio-thoracic care, including cardiac stents.” – Rajitha Senaratne, Minister of Health, Nutrition and Indigenous Medicine [47].

Not surprisingly, the 2018 UHC policy reaffirms the government’s support for “free” healthcare. Unique, however, are references to “free health” couched in the language of PPPs—specifically, public financing of private delivery. Under the strategic directions listed in the UHC policy document, 5.9 mentions “State recognition and regulation of Private Providers (Private General Practitioners), who can be purchased to provide health care *free* at the point of delivery to identified/opted persons,” and 5.13, “Effective engagement of the private sector and the involvement of the private General Practitioners in first contact care, ensuring provision of healthcare *free* at the point of delivery….” (emphasis added) (p. 4) [13].

This departure from public financing of *public* delivery is contentious. First and foremost, where will the money come from? Government health expenditure as a proportion of GDP is very low, at less than 2% [9]. Without market-oriented profit- and rent-seeking, and by adopting a strategy of over-subscription, the government has thus far controlled healthcare delivery costs through the public system. Given budgetary constraints, the Ministry of Health is rolling out the UHC policy incrementally, commencing with the development of a few strategically located primary-care centers [37]. Contracting-in private-sector services as planned in the next phases will invariably increase pressure on the health budget, particularly given the price differentials between public and private healthcare services [37, 38].

A second question is whether PPPs can address existing gaps in public healthcare, particularly the human resource and service deficits in remote and rural areas, without adverse impacts on the public system. Where implemented, PPPs have drained funds and other resources from public systems, without yielding the expected gains in equity or “efficiency” [48, 49]. In Sri Lanka too, contracting-in private services will divert much-needed resources from the public sector, weakening the very system that offers a semblance of UHC.

Contracting-in private providers may intensify human resource deficits in the public sector. At present, most private sector healthcare professionals in Sri Lanka hold on to public sector positions because of the security, benefits, and career advancement opportunities offered by the latter. This situation may change with the bolstering and expansion of private healthcare, as seen in India [48]. As private GPs—many of whom serve as medical officers at government hospitals—are contracted-in to work after hours at primary care centers, the availability of a fixed remuneration package financed by the government may drive at least some of these doctors to leave public service for full-time private practice. Similarly, contracting-in private pharmacy and laboratory services may result in a parallel weakening of these facilities in the public sector.

Despite its implications for the public system, a constellation of actors and forces favor PPPs as a health policy directive in Sri Lanka. In the domestic sphere, institutionalization of private interests within structures of governance has bestowed tremendous power in the hands of business. The government’s consistent failure to raise direct taxes—essential for mobilizing revenues for publicly financed and delivered UHC—reflects opposition by vested class interests [50]. Moreover, the state–healthcare industry nexus manifests starkly in the health policy domain. The government introduced a publicly financed health insurance scheme in 2017 to cover the private healthcare expenses of schoolchildren in spite of the availability of “free” healthcare and a functioning preventive health program in all schools [51]. Offering capped benefits for hospitalization, outpatient services (for specified chronic illnesses), and some accident/disability cover, the scheme was administered by a state-owned corporation in its first year [51]. Amid allegations of corruption, the government doubled the scheme’s benefits in 2019 and handed over its administration to a multinational insurance company [52].

Publicly financed health insurance or other forms of demand-side financing are imperative to expand the market for private healthcare. At present, the bulk of private healthcare is financed out-of-pocket, limiting its expansion to wealthier urban areas. Providing health insurance coverage or contracting-in private providers will undoubtedly raise levels of private healthcare use across the country. Moreover, separating healthcare purchasing from provision (“purchaser–provider split”) will enable public funds to be channeled for private profit as the government bankrolls healthcare expenditures, relying on private health insurance to carry out the purchasing function, generating massive profits for the industry [53].

The push for PPPs is implicitly supported by sections of the medical establishment. The Sri Lanka Medical Association (SLMA)—a professional medical body representing physicians in public and private sectors—has taken a leadership role in UHC advocacy. However, in promoting its vision for a “people-centered” healthcare delivery system, the SLMA has maintained a neutral stance on private delivery [54]. Meanwhile, the public-sector physicians’ union—otherwise vocal on all matters relevant to the health sector—has remained mute on the UHC reform. There is no reason to believe that the medical profession will hold out against efforts to integrate public and private healthcare delivery, because dual practice—an “informal” public–private arrangement—already constitutes a lucrative source of income for physicians [7, 55].

The Ministry of Health’s backing of PPPs may also be understood in light of its collaboration with the World Bank over the past two decades. Since 2000, the World Bank has rolled out three health-sector development projects in Sri Lanka. The first project (2003–2010), initiated during a cessation of hostilities between the government and the Tamil Tigers, was small in scope and scale. Although the project proposal contained plans for assessing the feasibility of alternative healthcare financing options [56], this was not followed through [57]. When the 30-year civil war ended in 2009, the incumbent government embraced a rhetoric of post-war development. The ensuing National Health Development Plan 2013–2017 [58], which coincided with the second World Bank-sponsored health-sector development project [59], laid out plans for PPPs, including a national health insurance scheme that was never implemented. The third (ongoing) World Bank health-sector development project, the “Primary Health Care System Strengthening Project,” was formulated in parallel with the 2018 UHC policy. The World Bank’s project appraisal report explicitly states that the proposed reforms will make way for a “public–private partnership enabling environment” (p. 22) [60], reflecting their ideological thrust.

Promoting public financing of private delivery towards achieving UHC in Sri Lanka goes hand-in-hand with the broader shift towards PPPs at the global level. International health and development agencies pay lip service to other avenues for financing UHC, such as raising taxes or increasing aid flows [61], but in practice, the focus has been on establishing PPPs, supposedly to “expand access to higher-quality health services by leveraging capital, managerial capacity, and knowhow from the private sector” (p.vi) [62]. The underlying impetus for this direction of health-sector development comes from global finance capital [63, 64]. UHC has opened avenues for accumulation for private healthcare and health insurance industries [65, 66] as well as a myriad of global health consultancies vying for the clientele of national governments [67]. Driven by profit-oriented rent-seeking, however, PPPs have been shown to perform poorly on equity [63, 68].

Given the support for PPPs at the global level, it is hardly surprising that the WHO has evaded the issue of public versus private delivery in its advocacy for UHC. On introducing UHC in 2010, it recommended national governments to “ensure that all providers, public and private, operate appropriately and attend to patients’ needs cost effectively and efficiently” (p. xviii) [61]. More recently, the 2018 Astana Declaration placed the responsibility for protecting the “right to health” with governments, but similarly skirted the issue of public or private delivery [69]. Supporting public financing without specifying a role for governments in health services delivery ignores the reality that bona fide universality, or even a semblance of it, has been achieved in low- and middle-income countries (LMICs) largely through publicly financed and delivered healthcare systems [70]. The WHO position today has served to mask (for-profit) private sector incursion and its consequences for healthcare systems and users in LMICs [53]—contrasting sharply with its championing of Health for All at the 1978 Alma Ata Conference, with universal *access* to be achieved by strengthening comprehensive primary care within “national health systems” [71]. Reverting to a vision of universal access that places people’s health and social justice aspirations at the center will require radical transformation at the global level.

## Conclusion

Sri Lanka’s publicly financed, administered, and delivered state-centric healthcare system has made critically important contributions to the country’s access achievements. Following decades of underinvestment and incentivized private-sector expansion, the public health sector is now struggling to meet the demand for services. The private health sector has stepped in to fill service gaps, but rising out-of-pocket expenditures are a major challenge, not least under the growing burden of NCDs in a rapidly aging population. The 2018 UHC policy is aimed at strengthening primary healthcare and providing access to an essential-services package through a mixed public–private “shared-care cluster system.”

Supported by the World Bank and others, the 2018 UHC policy addresses critical gaps in Sri Lanka’s healthcare system. Streamlining referral pathways can increase the utilization of primary-care facilities and reduce congestion at secondary- and tertiary-care centers. Implementing an essential services package should improve service comprehensiveness, population coverage, and financial risk protection. In addition to these well-devised strategies, however, there are also plans for formal partnerships with the private sector (PPPs) to advance UHC.

Does Sri Lanka need PPPs in the health sector? It is unclear how existing deficits in public healthcare can be addressed by PPPs. Expanding the role of the private sector in healthcare delivery will consume substantial public funds and channel scarce resources away from the ever-weakening public sector. Where implemented, PPPs have not delivered the expected gains in equity or “efficiency.” Instead of venturing into unexplored and potentially dangerous territory, the government would do well to inject more funds and strengthen the existing (and proven) model of UHC already operating in Sri Lanka. After all, the country’s access achievements have been largely due to this system.

PPPs are supported as a health sector development strategy for LMICs by a constellation of powerful actors and forces at the global level. Yet, UHC is a social goal that simply cannot be achieved by enabling profiteering and rent-seeking in the guise of expanding healthcare coverage. Re-envisioning UHC in ways that can meet people’s aspirations for health and social justice—that is the need of the moment.

## Data Availability

No datasets were generated or analyzed for the purposes of writing this commentary. Any data cited here may be accessed through the listed references.

## Abbreviations

GDP: Gross Domestic Product
GP: General Practitioner
IMF: International Monetary Fund
LMIC: Low- and middle-income countries
MCH: Maternal and Child Health
NCD: Non-Communicable Disease
PPP: Public-Private Partnership
SLMA: Sri Lanka Medical Association
UHC: Universal Health Coverage
USD: United States Dollar
WHO: World Health Organization

## Footnotes

1 This commentary draws on the author’s doctoral dissertation [7]

2 The Suriya Mal Movement later evolved into the Marxist Lanka Sama Samaja Party (1935) [17].

3 Fee-levying sections do exist in some public-sector tertiary-care centres.

4 A small proportion of seats at state universities are allocated to international students, on a fee-levying basis.
